# “I Prefer High-Intensity Exercise”—A Qualitative Study of Men’s Experiences with a Nature-Based Exercise Program for People with Arthritis

**DOI:** 10.3390/ijerph21121606

**Published:** 2024-11-30

**Authors:** Signe Andersson, Jonas Risum Ahler, Lars Hermann Tang, Thomas Vedste Aagaard, Søren T. Skou, Charlotte Simonÿ

**Affiliations:** 1 The Research and Implementation Unit PROgrez, Department of Physiotherapy and Occupational Therapy, Næstved-Slagelse-Ringsted Hospitals, Region Zealand, 4200 Slagelse, Denmark; jonae@regionsjaelland.dk (J.R.A.); larta@regionsjaelland.dk (L.H.T.); thva.regsj@gmail.com (T.V.A.); stskou@health.sdu.dk (S.T.S.); 2 Department of Regional Health Research, University of Southern Denmark, 5230 Odense, Denmark; 3 Center for Muscle and Joint Health, Department of Sports Science and Clinical Biomechanics, University of Southern Denmark, 5230 Odense, Denmark

**Keywords:** nature-based exercise, arthritis, user experience, qualitative research

## Abstract

Arthritis significantly reduces health-related quality of life, causing pain, fatigue, and decreased physical activity. To address this, exercise is highly recommended. However, men are less likely to participate in rehabilitation compared to women. We detected the same tendency in a nationwide nature-based exercise program in Denmark, with only 8% male participants. Therefore, this qualitative study investigated what engaged and restrained men with arthritis from participating in a nature-based exercise program. We employed interviews as the primary method for data collection. Data were analyzed using Braun and Clarke’s thematic analysis, revealing key patterns in participants’ experiences. This study finds that the fourteen participants’ experiences revealed two themes: (1) meeting with the nature-based exercise program was confusing, motivating, and disappointing, and (2) the social aspect is less important. To better engage men, future programs should include a clear description of the concept of the exercise, high-intensity exercise, a reduced emphasis on social activities, and consistency in the instructors’ roles and guidance. Incorporating these findings can better address the needs and preferences of men, helping them feel more like individuals than patients.

## 1. Background

Arthritis is a worldwide leading contributor to disability, and prevalence is only expected to rise dramatically over the next decades [1,2]. Around 162 million people suffer from musculoskeletal disorders, which, among others, include osteoarthritis (21.3 mil) and rheumatoid arthritis (3.08 mil). In total, 39% (62.9 mil) of those people who have musculoskeletal disorders are men [3,4,5].

Arthritis may reduce health-related quality of life, lower physical activity and function, cause disabling pain and fatigue, and increase the risk of additional diseases [6,7]. Physical activity and exercise are recommended treatments for arthritis across international guidelines [8,9]. However, a lack of ongoing motivation and long-term maintenance of physical activities often leads to difficulties following exercise-based recommendations for people with arthritis [10,11]. Furthermore, research indicates that men are less likely to participate in rehabilitation than women because they have a lower interest in health and do not find the professional help needed [12,13].

To facilitate the needed persistent behavioral changes, new and more user-friendly models are required [14,15]. One way is to introduce the nature setting into exercise offers. Nature-based exercise can be an alternative for people who do not feel their needs and preferences align with the traditional indoor setting [16,17]. According to reviews and register-based studies, nature-based exercise also can be an effective alternative and is, e.g., associated with a greater perception of personal health, reduced depression symptoms, increased energy level and enjoyment, and decreased risk of chronic diseases [18,19,20,21].

Therefore, The Danish Rheumatism Association initiated a nationwide nature-based exercise program in 2021, in collaboration with The Danish Gymnastic Association (DGI). This program is based on a previous DGI project for people living with anxiety, stress, and depression, offering an outdoor holistic exercise program with both physical and mental exercises [22]. The Danish Rheumatism Association adapted this program to people with arthritis while including activities to promote both physical and psychological well-being. The nature-based exercise program included exercises revolving around senses, strength, pulse, balance, games, and relations and was offered in 50 different geographic locations with one weekly exercise session within a 12-week period. Recruitment began in early 2022, and within two years, 1943 people participated in the project. However, men were largely underrepresented, with only 137 participants (8%). We already know that men are less likely to participate in rehabilitation, and that a nature setting could be an alternative to the more traditional indoor rehabilitation. A study from Knudsen et al. investigated the participants’ experiences with the nature-based exercise program, but men were largely underrepresented in the data collection. Therefore, we want to focus solely on the men’s lived experiences. To better accommodate the needs and preferences of men and ensure their participation in exercise-based interventions, a thorough investigation of the reasons for participating and not participating in exercise-based interventions is needed.

Therefore, this study aims to investigate what engages and restrains men from participating in the nature-based exercise program for people with arthritis.

## 2. Methods

### 2.1. Study Design and Philosophical Stance

This study is grounded in the phenomenological methodology [23]. Here, we try to understand how men experience the nature-based exercise program through conversations and interpretations of the spoken word [24]. We used an inductive qualitative approach by conducting individual semi-structured interviews [25] and analyzed them using Braun and Clarke’s six-phased descriptive thematic analysis technique [24]. The reporting adheres to the 32-item checklist for qualitative research from the consolidated criteria for reporting qualitative research (COREQ) [26]. The checklist is enclosed as File S1. The research team consists of four men and two women. The first author is a woman, new to research but experienced in physical activity. All of the other authors have solid experience in health research in exercise and either physiotherapy or nursing.

### 2.2. The Research Group

S.A. is an experienced physical activity instructor. T.A.A. is a physiotherapist and experienced researcher in complex interventions, diabetes complications, and exercise. J.R.A. is a physiotherapist and a Ph.D. student investigating the effects of the nature-based exercise program. L.H.T. is a physiotherapist and holds an associate lectureship in rehabilitation. S.T.S. is a physiotherapist and Professor of Exercise and Health. C.S. is a registered nurse who is experienced in qualitative research exploring patients’ perspectives and an associate professor in illness mastery and rehabilitation.

### 2.3. Participants and Recruitment

The Danish Rheumatism Association offered the nature-based exercise program and primarily aimed it at individuals with rheumatic diseases such as osteoarthritis or rheumatoid arthritis. However, there were no exclusion criteria, which also allowed participants without rheumatic diseases. Social and local media advertised the program, and participants were enrolled online or by phone. Moreover, hospitals, general practitioners, and primary care physiotherapists were informed about the program and encouraged to share the information with people with rheumatic diseases. The recruitment process in this study adhered to purposive sampling [27] among those participating in the nature-based exercise program.

We wanted to include participants who frequently attended the nature-based exercise (for more than six sessions) and participants who did not attend more than one to six sessions. We expected this would provide a more nuanced insight into the men’s experiences. Men who had participated in the Danish nature-based exercise program and responded to an outcome questionnaire were invited. Both men with and without any rheumatoid condition were invited to participate in this study. J.R.A., who was responsible for the questionnaires, mailed them with an invitation and a follow-up email if they did not respond. Fifty-four males were contacted, fifteen responded, with one declining to participate, and fourteen were included in this study. Table 1 provides the overall characteristics of the participants (*n* = 14). The mean age was 68.5 years (range 56–79), all were of Danish ethnicity, and most were retired (*n* = 10). Only one participant did not have a rheumatic disease or any pain in his back, joints, or muscles. Over half of the men suffered from comorbidities (*n* = 9). The first author, S.A., arranged a time and date for an interview via email, and everyone was offered a phone interview. No relationship was established with the participants before the interviews. After each interview, S.A. considered the saturation level, and by the eleventh interview on the 10th of November, no new angles were presented in the interviews. To be sure that saturation was reached, three more interviews were conducted, reaching a total of fourteen interviews. The participants attended the exercise program in either spring (*n* = 10) or autumn (*n* = 4) from 2022 to 2023 and were located nationwide in Denmark. There were no dropouts during interviews.

### 2.4. Setting

The Danish Rheumatic Association and DGI developed and delivered the nature-based exercise programs that began in the spring of 2022, with a new start-up in the autumn of the same year. This was repeated in 2023, with start-ups in both spring and autumn. Participants were charged 295 DKK to facilitate commitment and avoid many not attending the groups. Even though the concept was addressed to people with arthritis, it was not a criterion that you had to have a rheumatoid disease to participate. As recommended by the DGI, the exercise sessions were 60 min, once a week for 12 weeks, and available in 50 different geographic locations in Denmark. The instructor chose the nature setting, which included the beach, urban sites, the forest, and parking lots. The volunteer instructors had to participate in a two-day training course and were given a book with information and inspiration for possible exercises and program planning. The exercises presented in the book varied in intensity and addressed six parameters: senses, strength, pulse, balance, games, and relations. The instructors were also taught about health literacy, including how to handle participants dealing with pain, stress, anxiety, and terminal disease. Supporting health competencies and different teaching methods were also a part of the training course. Each session had up to 12 participants and included exercises like running up a hill, push-ups, and isometric wall squats, as well as exercises like collecting and touching different items, sensing the trees and the wind, or listening to the surroundings. The instructors were solely responsible for the 60 min of physical activity and had freedom in planning the program. Thus, they were required to adapt the intensity and exercise to the group and outdoor surroundings and could use the given book as inspiration.

### 2.5. Qualitative Rigor

Throughout this study, S.A. kept close contact with the co-authors. Overall, S.A. and C.S. were in close contact and are currently discussing the different parts of the study. In the process of developing an interview guide, we invited a user panel to help in clarifying possible themes. Because of the lack of male participation in the nature-based exercise program, men from another group-based exercise program targeting people with arthritis were chosen. Hence, three men participating in Good Life with osteoArthritis in Denmark (GLA:D), which is an indoor structured exercise therapy, were invited to talk about their experiences with the GLA:D concept [28]. A physiotherapist who worked with the GLA:D concept was contacted and asked if any of his male participants would be interested in participating in a user panel for this study. Three men agreed to be contacted by S.A. The purpose of the meeting was to learn about the men’s experience with participating in a structured exercise concept, to talk about what they valued, and to talk about their immediate thoughts on the nature-based exercise program. They were told that we wanted to improve the nature-based exercise program to include more male participants. This meeting was one hour, took place at the research unit PROgrez, and was arranged by S.A. and T.A.A. The interview guide was then adjusted to contain some of the themes from the meeting.

Before the analysis was made, S.A. and T.A.A. discussed the methods and how to follow the six phases by Braun and Clarke [24]. On several occasions, possible topics for the discussion and the findings from the analysis were discussed with all co-authors.

### 2.6. Data Collection

The interview guide focused on three main questions with matching sub-questions. The full interview guide is enclosed as File S2.

1. Did you know what you were getting into before you started the nature-based exercise program?
2. Describe what made you continue to participate/withdraw from the program.
3. Can you provide some suggestions on how we can make future offers more appealing to men?

S.A. conducted one interview with each participant on the phone. Because of the geographical spread, phone interviews were chosen as the primary interview method [29]. This meant that the participants could choose their location for the interview. Some were at home, some had their partner in the background, and one was walking through the forest. One participant required an online meeting, and one wished for the possibility to write his answers down and have a short follow-up phone call afterward. This interview was recorded and transcribed like the others. Both written answers and the transcription were included in the coding. Before the interview began, the participants were reminded that the interview would be about their experience with the nature-based exercise program, that they should be as honest as possible, and that we would like to gain some perspective on how to attract more male participants in the future. S.A. also mentioned that the interview was being recorded. S.A. had prepared some open questions and was focused on having a free-flowing conversation. Sometimes, S.A. repeated a question from the interview guide to make sure the participant answered. If the participant talked about something not relevant, S.A. steered the conversation back on topic. They did not receive information about the interviewer before the interview, but some asked about education and occupation during the closing questions. The duration of the interviews varied between 33 and 63 min, except for the follow-up phone call, which lasted 3 min. All interviews were recorded on a Dictaphone and stored in a secured database. S.A. took notes during the interviews. These were later properly disposed of. A true verbatim transcription was conducted by S.A., and all meanings were ensured. Participants did not read or comment on the transcriptions. Data collection took place in October and November 2023.

### 2.7. Data Analysis

Data were analyzed using Braun and Clarke’s six phases in the thematic analysis [24].

**Figure 1:**
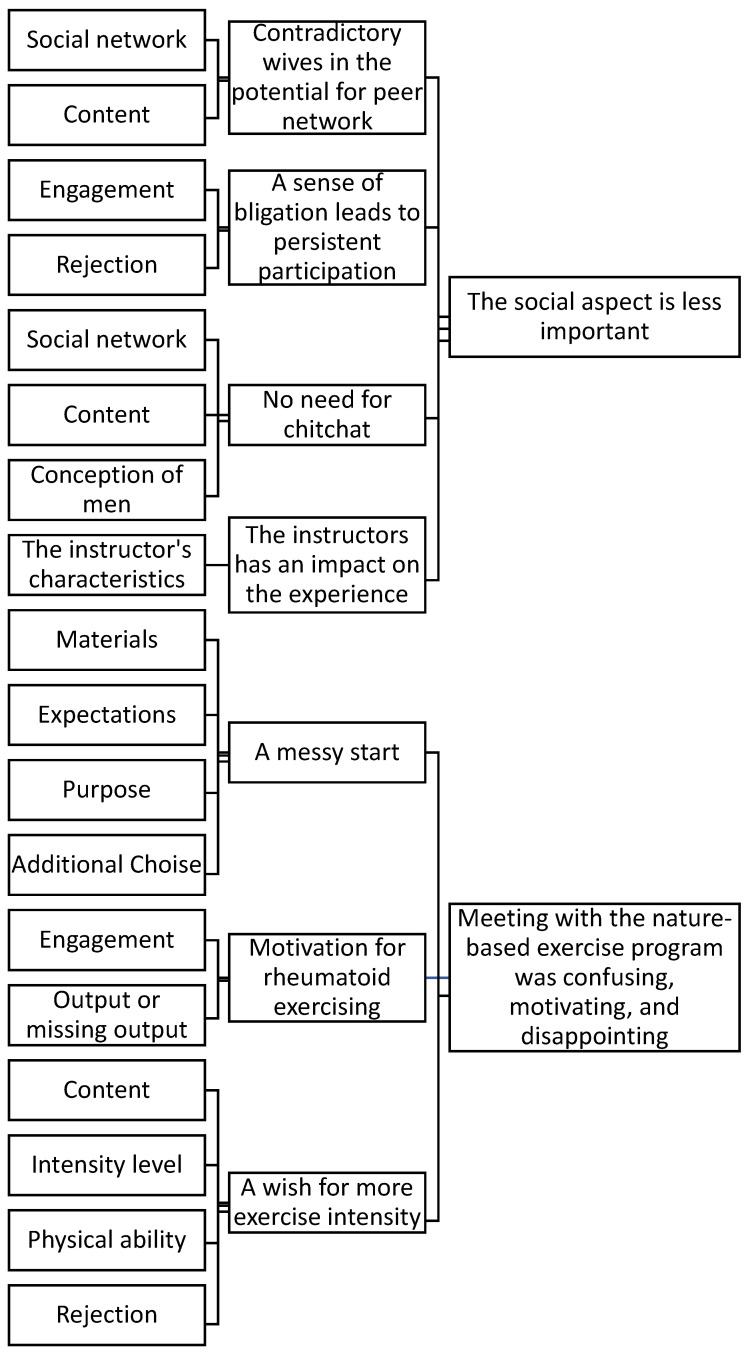
Coding tree.

In the first phase, SA familiarized with the data by reading the transcribed interviews to gain an overall understanding of the data. In phase two, initial codes were created based on more descriptive semantic coding. All codes were connected to specific sentences in the transcription, giving a large diversity of initial codes. S.A., T.A., F.B.A., and C.S. collaborated on the coding here. The initial themes were developed in the third phase by exploring the initial codes and connecting them into possible themes. The codes were divided into different groups, dissolved, and rearranged into new constellations. The initial themes were based on the overall subject of the codes, and in the following phase four, the initial themes were formed into more extensive and more nuanced themes in collaboration with C.S. Afterward, S.A. worked on defining and naming the themes as Braun and Clarke described in phase five. The themes were written down, presented, and discussed in phase six with the co-authors. This led to a revisit to phases four and five and a new constellation of the initial codes. This was performed several times until the co-authors agreed on the final themes [24]. Figure 1 shows the final coding tree. Participants did not comment on the analysis.

The study was carefully discussed through debriefing, member checking, and reflexive journaling within the author group to obtain confirmability. The whole author group undertook the six-phased thematic analysis following the phases of thematic analysis to ensure credibility in the findings. Selected quotes are included in the description of the findings to provide transparency [24]. Moreover, transferability is strived for in the discussion [30].

### 2.8. Ethical Consideration

This study was conducted with the approval of Region Zealand’s Ethics Committee on Health Research (EMN-2021-09728), The Danish Data Protection Agency (REG-147-2021), and the Helsinki Declaration [31]. All participants received written information about the purpose and the method of the study before deciding if they wanted to participate. Only the ones who previously had agreed to be contacted received an invitation. Before the phone interviews were conducted, S.A. reminded the participants about the topic and that the conversation would be recorded. All participants signed a declaration of consent, with the possibility of withdrawing at any time.

## 3. Findings

Ten of the fourteen participants had participated in more than six sessions in the nature-based exercise program. Four participated in four to six sessions.

The analysis revealed two primary themes concerning the unsureness regarding the overall purpose of the concept and their absent need for socializing. These themes were based on the thematic analysis and reflected on what engaged and restrained men when participating in the nature-based exercise program, as shown in Figure 1.

The coding tree shows the coding during the analysis, moving from initial codes to final themes. Some initial codes are incorporated into more than one theme.

## 4. Meeting with the Nature-Based Exercise Program Was Confusing, Motivating, and Disappointing

### 4.1. A Messy Start

The men in this study chose to participate in the nature-based exercise program for three reasons: because they wanted to spend time in nature, they wanted to exercise, or a combination of both. Some explained how they sought out an exercise concept by themselves, while others had it requested by their family or partner. The majority needed a push to get started, either by themselves or by someone close to them, as one of them explained,

*“It was my daughter who sent me the information about the concept and nudged me… no, not that she kicked me… I looked at it and saw it as an interesting opportunity.”*

(participant 5)

Entering the nature-based exercise program appeared confusing to men. They experienced a messy start because the purpose of the nature-based exercise program was uncertain to them. They disclosed being in doubt of what they are a part of. They expressed confusion concerning the target group of the nature-based exercise program and were unsure whether it addressed people with arthritis or if people without any physical discomfort could join. However, they tended to create their own purpose for participating, like getting some exercise, taking care of themselves, or, as one said,

*“I wanted to get a bit of inspiration for what to do in everyday life to get ongoing, and be a bit more active, and to keep the disease at a distance.”*

(participant 9)

The interviews show the men experiencing a mismatch between their expectations and the actual content of the nature-based exercise program, as one stated: *“I expected to get sweatier. Not much but a bit.”* (participant 4)

### 4.2. Motivation for Rheumatoid Exercising

Nevertheless, while being in the nature-based exercise program, they discovered new perspectives, as described by the same participant: *“However, it is not necessary to sweat. You must get your ankles and arms thoroughly worked out and things like that. I think that exercise for people with rheumatism is to make you move the different parts of your body.”* (participant 4)

Furthermore, some described being positively surprised by the nature-based exercise program. They were happy with the content and variation of the exercises and were left with a feeling of a clear conscience. The men were not concerned about the season and the weather conditions. They were positive about being outside and mentioned wearing warmer or waterproof clothing if the weather was bad. The only problem with the seasons was that it often became dark in the evening in the autumn, and it was difficult to see unless they had a headlamp or flashlight.

The men disclosed how they look at the nature-based exercise program as a facilitator of being physically active and thus motivating. One said,

*“We were not there to support each other in feeling bad. We came to support each other to exercise, to get outside, and to be with other people.”*

(participant 11)

### 4.3. A Wish for More Exercise Intensity

Unfortunately, several experiences showed that the current content of the nature-based exercise program did not fulfill their expectations and own purposes. Accordingly, it was viewed as disappointing. They described that the exercises lacked intensity and were not challenging enough regarding their physical shape. In addition, they explained that exercises focusing on mental or playful aspects could be reduced or left out entirely. One said,

*“I could have given better and clearer feedback to our instructor, explaining that I expected to sweat, right, to reach and hit the pulse limit. At least as a man, that was a huge expectation, and sometimes the instruction became too playful, right.”*

(participant 7)

Several men stated that they tried to push themselves as much as possible during the exercises, when feasible, as shown in the following description:

*“Well, I like to be outside, and I thought, even though it was not as intensive as I hoped, you can work on making it as intensive as possible by yourself. And I did that.”*

(participant 11)

Even though the men could choose how much they wanted to push themselves during the exercises, they still did not feel the content met their expectations. One described the possibility of adjustment as follows:

*“There might come a time when you are incapable of doing much, but then you can turn down the level of the exercises. In this program, I thought the level was too low to begin with, so it could not be turned down any lower.”*

(participant 9)

The men understood that everybody could participate no matter their physical state. Those who were less impaired by arthritis or other physical limitations tended to turn down the offer of another exercise program to favor those who were more affected, as one stated:

*“… but still, I need to raise my pulse and such when I exercise, right? So, it would still not be fulfilled by the concept you have now. It is properly not what you and the Danish Rheumatism Association need to focus on. It should properly be the ones with problems, I think.”*

(participant 8)

As shown in the quotation above, the current nature-based exercise program was not challenging enough for several participants. They wanted to find another option that met their needs and did not want to participate again with the current design of the nature-based exercise program.

## 5. The Social Aspect Is Less Important

### 5.1. Contradictory Wives on the Potential for Peer Network

The men considered the opportunity to meet and exercise with peers differently. Some described that the chance of being with others encouraged them, as one stated: *“As in my case, being xx years of age, I am retired, and thus I miss somehow being social.”* (participant 14)

On the contrary, most men experienced exercise sessions to focus too much on socializing and small talking. One depicted, with explicit reference to the women from the group, *“It seemed that some participated, mostly to have someone to talk to and walk with. In my opinion, I attend to do some efficient exercises. If it were a matter of taking a walk, I would rather do that back home.”* (participant 12)

Moreover, they described that the group-based exercises tended to induce indifference rather than being relevant.

### 5.2. A Sense of Obligation Leads to Persistent Participation

The majority of the men stated that they kept participating due to feelings of obligation, whereas a few mentioned a social commitment towards peers or the instructor. The prime reason for their persistent participation relied upon a sense of taking the opportunity from someone else and a mindset that if you choose to participate in something, you do not quit. Therefore, they decided to continue, as one explained:

*“I tried to be there every time, even though I quickly realized I did not want to continue afterward, but I thought, I could just as well carry through, right.”*

(participant 9)

Even though the men did not see their expectations for exercise met, they kept participating because they found it manageable to accomplish. They perceived the nature-based exercise program as relatively short and less demanding, as one explained:

*“I might have chosen to come anyway just to see what it was, right? As I say, it was not a huge investment to throw oneself into, and it was not because it was something that, regarding time, interfered with your everyday life.”*

(participant 9)

### 5.3. No Need for Chitchat

The men described that the social aspect took too much time instead of physical activity and sometimes seemed disturbing. In some cases, some deliberately chose to withdraw from socializing during and after the exercise session.

Some explained that they wished for more male participants because they experienced the conversations to be quite female-oriented. They disclosed that being with more men might have been better, as one described:

*“I think the social aspect will be different. It would be easier, with a male talk […] You sometimes feel that you interrupt because many of them talked with each other, and they quickly formed some groups.”*

(participant 3)

These men also reflected that an increased number of male participants would cause a better social dynamic and increase the intensity of the physical exercises. Others mentioned that they will continue their current social behavior with or without the presence of more men.

### 5.4. The Instructor Has an Impact on the Experience

The men experienced the instructors in very different ways, but in general, they described them as engaged, prepared, and kind. In some cases, the men easily related to the instructor who was also having difficulties with physical movement, as one explained:

*“Well, she talked about her progress. She had many problems with her body, and she did a lot of physical workouts, ran, etc. That was how she was able to inspire us, which I thought was very good.”*

(participant 1)

The men viewed the instructors’ prioritization of exercises differently. Some instructors focused on storytelling, while others prioritized more physical exercises. In some cases, men with more than one instructor were satisfied with two very different approaches, while others appreciated one better.

Several men mentioned the instructors’ voluntary work. They greatly respect people who choose to spend their leisure time as an instructor. On the other hand, they mentioned that it will make a positive difference if the instructors are professional. One described the variety in this way:

*“The first one exercised himself, and then he took a course. You cannot say that he was a professional. However, the one we have now is an occupational therapist or something like that. Anyhow, she knows something about different types of motion and such stuff. It is also harder and more concentrated.”*

(participant 10)

## 6. Discussion

This study found two main themes concerning what engaged and restrained men from participating in a holistic nature-based exercise program. The themes showed that men engaging in physical activity were focused on being as active as possible. They valued a high level of intensity over the social aspect of the nature-based exercise program. The men needed a clear description of the overall nature-based exercise program because, otherwise, they might experience a divergence between their expectations and the content of the exercise sessions.

### 6.1. A Holistic Approach to Nature-Based Exercise

Previous research on nature-based physical exercise covers a wide range of different activities and encompasses everything from structured nature-based physical exercise, focusing on strength and condition, to holistic activities like experiencing nature through sitting, social and therapeutic horticultural activities, and nature-based arts and crafts [32,33,34,35,36]. The nature-based exercise program, developed by The Danish Rheumatism Association, focused on a more holistic use of nature. Our study showed that the men were focused on being as physically active as possible, and they expressed their wish for more intense exercise and less with a sensory focus. Because of the major differences in the distribution between males and females, the more holistic approach may have appealed more to women. A study shows that men are less likely to participate if they find the exercise offer too “feminine” or if they experience gender inclusivity or an absence of homogeneity [37]. Therefore, if the current nature-based exercise program should appeal to more men, it might have to deviate from the holistic approach and look more towards using nature as structured nature-based physical exercise.

Keniger et al. describe three different degrees of natural involvement in exercise programs: indirect, incidental, or intentional interactions. Indirect interaction is often used in an indoor environment, with some elements of visual nature on video monitors or virtual-reality glasses. An incidental interaction is when physical exercise, which normally takes place in indoor surroundings, is moved outdoors. An intentional interaction is when the patient intentionally engages in nature, like gardening, hiking, climbing hills, and so on [38]. The nature-based exercise program contained incidental interactions like icebreakers at the beginning of the first exercise sessions or when instructors used elastics or yoga mats to do exercises without the direct involvement of nature. Most of the exercises had an intentional interaction with nature, such as collecting and using sticks and other items, running between trees in the forest, or lifting different objects. Our study identified that men enjoyed exercises such as running up a hill and lifting stones or heavy branches. They focused more on vigorous exercise and how nature could be included in fulfilling their expectations rather than finding leaves, collecting pinecones, or touching trees. Therefore, more exercises focusing on strength and physical fitness can be added to the intentional interactions to adapt to the preferences of men.

In the review from Gavarkovs et al., men with arthritis are described as less likely to participate in rehabilitation because they have difficulty addressing their illness, they do not feel sick enough to participate, or arthritis has not yet had an impact on their work function or daily activities [39]. In our study, some men participated because of their arthritis, but all wanted to exercise and did not feel the need to engage in conversations about living with arthritis. Thus, the nature-based exercise program has the potential to appeal to men because it is likely to position them as healthy humans.

### 6.2. Preferences and Fulfilling the Participants’ Needs

The men in our study experienced unclearness regarding the purpose of the nature-based exercise program, and many did not feel that their expectations were being met. The program content is an important factor in the participant’s desire to attend [37], and not getting one’s expectations and wishes fulfilled negatively influences the participant’s motivation [40]. Knowing the purpose of the exercise program also influences the participant’s effort, attention, and strategizing toward reaching the goal [41]. Therefore, it seems essential that the purpose of the nature-based exercise program is made clear so the participants know what to expect and are motivated to keep participating. Because the participants also expressed confusion regarding the target group for this exercise concept, the organizers could clarify to whom the nature-based exercise program is addressed. It must be clear to avoid confusion, whether they only want to include people with rheumatoid disease or everybody who wants to exercise in nature.

In our study, more intense exercises were the most common wish of the participants, and many felt their expectations for this specific topic were unfulfilled by the current nature-based exercise program. As pointed out in the study from Poveda-López et al., both men and women have a wish for exercises that require the right amount of intensity and effort, that are adaptable and not too childish [42]. The same is evident in our study. The nature-based exercise program needs to have more adaptable exercises where participants can obtain the intensity and put in the amount of effort they choose. Removing the more playful exercises may also make the nature-based exercise program more appealing to future participants.

Our findings reveal that the instructor plays a big part in men’s experience when attending the nature-based exercise program. They are responsible for choosing the location and planning each exercise session. A study by Windt et al. also shows the importance of the instructor. The elderly male participants especially valued an instructor with exercise experience, who was used to working with elderly adults, could take criticism, and offered an individualized and fun exercise program [37]. Because the instructors in the nature-based exercise program are volunteers, it cannot be expected that they possess the same qualities as requested in the study by Windt et al. Training the volunteers is very important to ensure they carry out their role [43]. Furthermore, studies show that participants in exercise programs prefer professional physiotherapists with expertise in arthritis [44,45]. If the instructors in the nature-based exercise program should be able to accommodate some of the changes, e.g., the ability to adapt the intensity of the exercises and ensure the exercise sessions comply with the overall purpose, they will need sufficient training, or the inclusion professional physiotherapists must be considered.

### 6.3. The Social Aspect Is Not as Important to Men

Our findings show that many male participants did not prioritize social interaction during the nature-based exercise program. Several expressed that they found the social aspect too dominant compared to the number of physical exercises. Some engaged in the social aspect but did not consider it as a reason for their participation. Others completely withdrew from social interaction and preferred focusing only on the exercise. Instead, a large portion of the men continued to participate due to a sense of obligation because once they sign up for something, they feel the need to complete it.

Our findings, however, differ from previous studies showing that social engagement generally includes a sense of belonging to a group and experiencing a social connection, which are important factors in an individual’s motivation to engage in physical activity [46]. The social element in exercise is reported to significantly influence elderly male participants’ motivation for ongoing physical activity behavior [47]. The men in our study do not tend to feel motivated by the social element to the same extent, and therefore, it is not considered a significant reason for their decision to participate and their continued participation in the nature-based exercise program. One reason might be that the social aspect of the current nature-based exercise program is influenced by the fact that very few men participate.

Instead, men report a high sense of obligation to fulfill the program, which is also found in another study by Knudsen et al. This study is based on the same overall nature-based exercise program as ours, but the two studies are different in structure and are independent of each other. The study by Knudsen et al. is based on qualitative interviews with twelve women and three men, and it shows a different perspective on social and more playful activities in the exercise sessions. It describes how the program contributes to creating a more playful atmosphere and provides a safe space for participants as they become more comfortable with the group and want to try new things [48]. This was not mentioned by the men in our study. On the contrary, our study found men wishing to avoid the playful exercises and instead focus more on intensity and structure. If the goal is to encourage more men to participate, it might be an option to shift the focus away from social exercises.

In summary, deviating from the more holistic approach may be necessary if the goal is to recruit more male participants to attend future nature-based exercise programs. The purpose needs to be clear and correspond with the actual program scheduled by the instructor. The program needs to include more intensity with the possibility of regulating by progressing or regressing the exercises. The more playful and mental exercises can be left out or reduced. The men did not choose the nature-based exercise program because of social aspects, and the focus should be on physical exercises and not on creating a social community.

### 6.4. Strengths and Limitations

Among the strengths of this study is that 14 men with varied sociodemographic and health profiles were included. Hence, they represent different geographic areas in Denmark, resulting in a wide range of experiences based on various instructors’ differing content in the exercise program. Another strength is that the instructors had considerable freedom to shape the exercises. Hence, the findings cover a broad span of the overall Danish nature-based exercise offer.

It can be considered a limitation that the participants attended four to twelve sessions and thus were likely to appreciate the program. Our initial plan was to include an equal number of participants who participated more than six or fewer times. However, during the data collection, it became clear that the degree of participation did not necessarily reflect the men’s opinion towards the nature-based exercise program. Therefore, the recruitment stopped when saturation, regarding the aim, reached fourteen participants [25]. Nevertheless, it is a limitation that not all points of view are represented in this study. Men with early dropout within the first four sessions of the program are not represented in this study. The perspective of those men could add nuance to the findings. Further studies can help us understand why, e.g., men do not choose to participate in the program.

To our knowledge, we deem that this is the first investigation of the experience of nature-based exercise programs from a solely male perspective, which should be considered a significant strength. Given that only 8% of the participants were male, the conclusion should be considered with this in mind. This study should be followed by new research investigating the male perspective in other exercise programs.

## 7. Conclusions

In conclusion, we found that men with various arthritis diagnoses chose to participate in the Danish nature-based exercise program because they wanted to be physically active. Their primary focus is on the intensity of the exercises. On the contrary, men tend not to value engaging in social activities without a clear exercise outcome. In future nature-based exercise programs, the male-appealing potential of positioning participants as more healthy humans than as patients must be considered. To address the men’s needs, a clear description of the purpose of the exercise concept and the inclusion of intentional interactions with high-intensity exercise is important. Less focus on social activities is recommended. Moreover, alignment in instructors’ roles and guidance, e.g., guiding on individual levels, can improve the program. The results can inform future programs to address the needs and preferences of men better and thus potentially improve the positive outcomes of being physically active in this growing population.

## Figures and Tables

**Table 1:** Participant characteristics.

	Variable	Number of Participants
Socio-demographics	**Living status**	
	Alone	3
	Together	11
	**Highest education**	
	Vocational education	4
	Short-cycle higher education	2
	Medium-cycle higher education	6
	Long-cycle higher education	2
	**Occupation**	
	Retired	10
	Ordinary work	3
	Other	1
Health	**Arthritis or pain condition (possibility for multiple choices)**	
	Osteoarthritis	7
	Rheumatoid arthritis	7
	Polymyalgia rheumatica	2
	Spinal stenosis	1
	Non-specific low back pain	2
	Gout	2
	Pain in muscles and joints but no arthritis	1
	Osteoporosis	1
	Vertebral compression fracture	1
	No arthritis-related disease or pain	1
	**Other comorbidities (possibility for multiple choices)**	
	No other diseases	5
	Other diseases that arthritis	9
	**First time feeling functional limitation or pain in muscles or joints**	
	Under 10 years	8
	10 or more years	5
	No functional limitations	1
	**Pain level the latest week, 0–100 on the Visual Analogue Scale (VAS)**	
	Under 50	8
	50 or over	6
	**Participation in physical activity with an instructor or physiotherapist within the last three months**	
	Yes	3
	No	11
	**Physical activity level, 1–10 on the UCLA Activity Scale**	
	Light physical activity (1–4)	4
	Moderate physical activity (5–6)	7
	Vigorous physical activity (7–10)	3
	**Frequency in participation in physical activity leading to sweating or being out of breath**	
	Once a week or less	6
	More than once a week	8

The table shows participants’ sociodemographic and health profiles.

## Data Availability

Due to ethical rules the data of this study is not shared.

## Supplementary Materials

The following supporting information can be downloaded at: https://www.mdpi.com/article/10.3390/ijerph21121606/s1, File S1: COREQ Checklist, File S2: Interview guide.
