# Supplementary material for: “I Prefer High-Intensity Exercise”—A Qualitative Study of Men’s Experiences with a Nature-Based Exercise Program for People with Arthritis
Source: Int J Environ Res Public Health. 2024 Nov 30;21(12):1606. doi: 10.3390/ijerph21121606 (PMC11675972; doi:10.3390/ijerph21121606)
Supplement: Supplementary file 1 [file ijerph-21-01606-s001.zip › File S2 - Interview guide.pdf]

Interview guide

Main questions	Sub questions
Did you know what you were getting into before you started the nature-based exercise program?	- How did you hear about the nature-based exercise program?<br>- What attracted you to the nature-based exercise program?<br>- What were important for you to know before you started?<br>- Did you feel that you had received sufficient information before you started?<br>- Where you skeptic or worried about anything before you started?<br>- What were your expectations for the first exercise session?<br>- Did the exercise session meet your expectations?<br>- Did you mainly participate because of your arthritis?
How many times did you participate in the exercise sessions?	- How was it distributed?
Describe what made you continue to participate/withdraw from the program?	- Did the weather influence your participation?<br>- Did you do something new every time, or did you know in advance what was going to happen? And how did you feel about that?<br>- Did you experience any progress during the program e.g. improved mobility or better balance?<br>- How was the distribution of men and women in your group? Did it influence your wish to participate?<br>- What time of day was the exercise session? Did the time of day suit you?<br>- Did you feel that you had been physically active when you return from the exercise sessions?<br>- Was it important to you that the exercise was intense, that you pushed yourself and got sweaty? Did you achieve that during the exercise sessions?<br>- How was the distribution of the different exercises?

Can you provide me with some suggestions on how we can make future offers more appealing to men?	- If you were offered another nature-based exercise program in the nearest future, would you participate?<br>- What would make you want to/not want to participate?<br>- Are there anything that could be changed to make you more inclined to participate?
Closing questions	- Are there anything I have forgot to ask you about?<br>- Do you have any questions?
